# Theoretical and Empirical Basis of Psychogenic Aging

**DOI:** 10.1192/j.eurpsy.2025.318

**Published:** 2025-08-26

**Authors:** M. Faria

**Affiliations:** 1 Cognition and Brain Sciences Unit, University of Cambridge, Cambridge, United Kingdom

## Abstract

**Introduction:**

Psychogenic aging, an emerging field in biogerontology, seeks to integrate psychological factors into the biological framework of aging. Traditional models of aging emphasize molecular and cellular mechanisms, often overlooking the significant role of psychological well-being. Our studies show that psychosocial factors influence biological age similarly to physical health conditions. Psychogenic aging is a novel phenomenon that addresses this gap by providing a holistic understanding of the aging process that encompasses both biological and psychological components.

**Objectives:**

The primary objective of this research is to explore the relationship between psychological factors and biological aging. Specifically, it seeks to quantify the impact of psychosocial stressors and well-being on biological age and compare these effects with established biological markers of aging. This study also aims to position psychogenic aging within the established Hallmarks of Aging framework.

**Methods:**

Data from the China Health and Retirement Longitudinal Study (CHARLS), including 16 blood biomarkers and psychological well-being indicators for over 11,000 adults, were used to train a deep neural network (DNN) to predict biological age.Psychosocial factors such as happiness and loneliness were regressed against biological age using elastic net regression to quantify their effects.

Additional findings from a non-systematic review on stress and aging were used to frame the psychogenic aging model within the Hallmarks of Aging framework by Lopez-Otín *et al.* 2013 Cell.

**Results:**

Galkin *et al*. Aging 2022 developed a deep-learning aging clock using blood test data to assess the influence of psychological factors on biological aging. The results show that negative psychological factors, such as loneliness and unhappiness, can accelerate biological age by 1.65 years, surpassing the effect of smoking, which contributes an additional 1.25 years. These findings highlight the substantial impact of mental well-being on biological age, comparable to serious health conditions like stroke and lung disease. Faria *et al.* Trans Psych 2024 developed a theoretical framework from which to conceptualize the mechanisms and mediators behind these psychogenic aging effects with the latest high-throughput methods.

**Image 1:**

**Figure:**
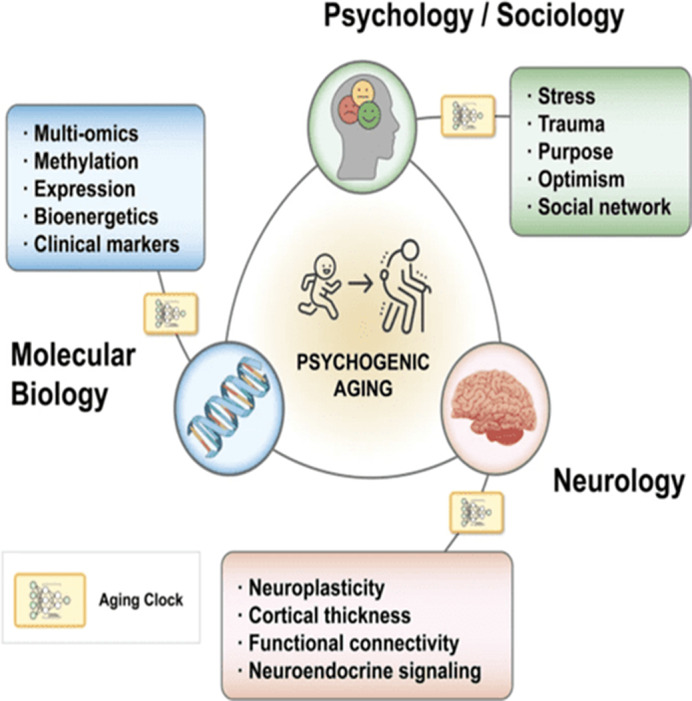

**Image 2:**

**Figure:**
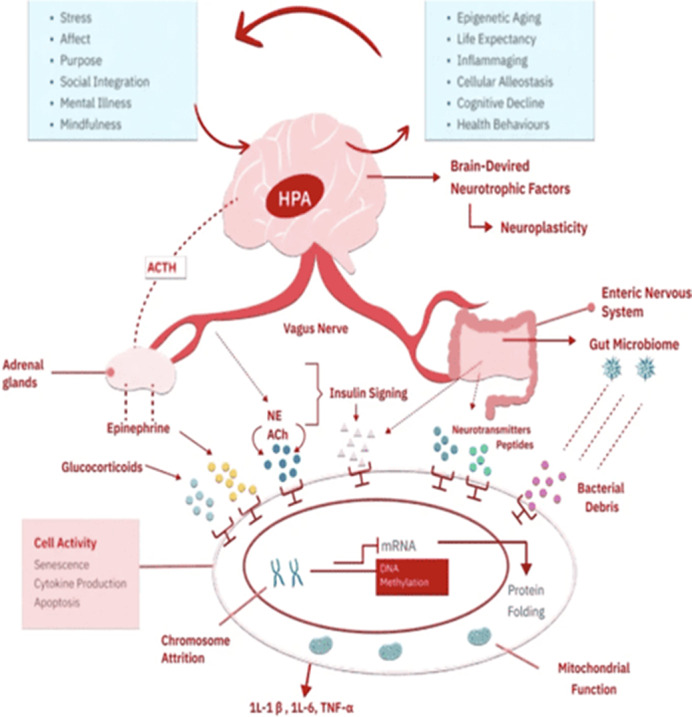

**Image 3:**

**Figure:**
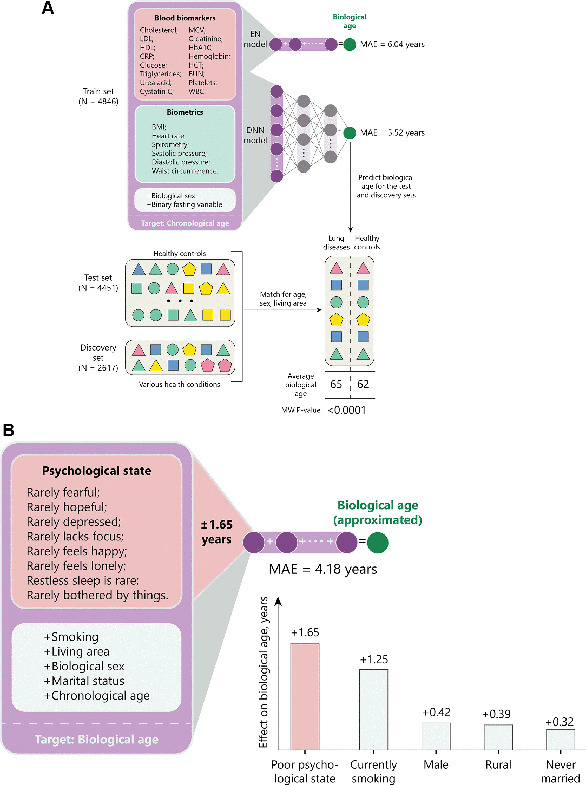

**Conclusions:**

The integration of psychogenic aging into the Hallmarks of Aging framework represents one of the most significant advancement in our understanding of the aging process. Psychological stressors and resilience should be recognized as fundamental contributors to aging, akin to molecular and cellular processes. By addressing both the biological and psychological dimensions of aging, this interdisciplinary approach offers novel opportunities for anti-aging therapies and interventions. Future research should focus on validating these findings across diverse populations and exploring the mechanistic pathways linking psychological health to biological aging.

**Disclosure of Interest:**

None Declared

